# Seasonal Changes of Nuclear DNA Fragmentation in Boar Spermatozoa in Spain

**DOI:** 10.3390/ani11020465

**Published:** 2021-02-09

**Authors:** Raquel Ausejo, Juan Manuel Martínez, Pedro Soler-Llorens, Alfonso Bolarín, Teresa Tejedor, María Victoria Falceto

**Affiliations:** 1 Department of Animal Pathology, Obstetrics and Reproduction, Faculty of Veterinary Medicine, University of Zaragoza, 50013 Zaragoza, Spain; sveterinarios@magapor.com (R.A.); abolg2000@hotmail.com (A.B.); vfalceto@unizar.es (M.V.F.); 2 Department of Biotechnology R&D, Magapor S.L., 50600 Ejea de los Caballeros, Spain; biotecnologia@magapor.com; 3 Department of Research and Development, Magapor A.I.E., 50013 Zaragoza, Spain; 4 Department of Anatomy, Embryology and Genetics, CIBERCV, Faculty of Veterinary Medicine, University of Zaragoza, 50013 Zaragoza, Spain; ttejedor@unizar.es

**Keywords:** photoperiod, boar, sperm, DNA fragmentation, spermatogenesis

## Abstract

**Simple Summary:**

Artificial insemination is widely used in pig production and currently a boar performs several thousand matings per year. Traditionally sperm quality is focused on the number of spermatozoa, their motility and morphology. However, the quality of sperm DNA, which contains genetic information, is also related to fertility problems. The aim of this research was to study the effect of natural light hours and age of the boar on the status of the sperm DNA. After a powerful statistical analysis, it was found that the percentage of spermatozoa with fragmented DNA decreases within the observed age range as the boar gets older. On the other hand, the amount of spermatozoa with fragmented DNA was the lowest in autumn while it was the highest in summer. This study demonstrates the remaining seasonality of boars in Spain and highlights the importance of controlling the environmental conditions in the farms. Sperm DNA testing provides a basis for improving the selection of AI boars by excluding males with higher DNA fragmentation due to their very young reproductive age that may pose a potential subfertility.

**Abstract:**

There are numerous cases when conventional spermiogram parameters are all within an acceptable range but boar subfertility persists. The total sperm nuclear DNA fragmentation index (tDFI) is a trait related to fertility and prolificacy problems that is not routinely evaluated in commercial AI boars. The aim of this research was to study the effect of the photoperiod, season and reproductive age of the boar on tDFI (measured by SCSA) of 1279 ejaculates from 372 different boars belonging to 6 different breeds located in 6 AI studs in Spain. tDFI data ranged from 0.018% to 20.1%. Although there was a significant single boar effect in the tDFI occurrence, a negative correlation between the tDFI and the age of the boar was found (*p* < 0.001). tDFI would decrease due to aging of the boar 0.66% each year old within the observed age range. After including age as a covariate in the ANCOVA, no differences were found in tDFI between photoperiods when the sperm collection date was evaluated. However, when the date of the production of semen in the testis was evaluated, the total percentage of spermatozoa with fragmented nuclear DNA was 1.46% higher in the increasing photoperiod in comparison to the decreasing photoperiod (*p* < 0.0001). On the other hand, for both dates, the lowest tDFI values corresponded to minimum day length for decreasing photoperiod phase (autumn), while the highest tDFI values were found in summer (maximum day length for decreasing photoperiod phase).

## 1. Introduction

The competitiveness of industrial pig production depends, among other factors, on the reproductive output that is governed by many elements affecting both females and males. There is a large variation in the fertility results, mainly caused by farm- and sow-related parameters (reviewed by Knox, 2016), and artificial insemination (AI), the most important tool used to spread the genetic improvement in the pig industry [1,2,3]. Based on the current experience of the boar AI centers in Spain (AIM Ibérica (Topigs Norsvin Spain), personal communication), 2000 sows and/or gilts are being inseminated with ejaculates from just one boar. Nevertheless, the aim of 6000 inseminations per year with one boar’s ejaculates is an expectation not yet accomplished (reviewed in Roca et al., 2016 [4]). The conventional approach for evaluating sperm quality is mainly focused on sperm motility, the total count of sperm cells in the dose and sperm morphology [5]. These parameters have been traditionally associated with male fertility. When sperm motility or morphology are poor, the boar is no longer considered as a valid breeding animal, or the number of sperm cells in the AI dose is increased in the commercial AI dose. Nevertheless, there are some semen traits affecting fertility that are not compensable by adding more sperm cells in the AI dose [6] or they are not identified in the conventional spermiogram [4] thus occurring at a molecular level [7].

However, there are numerous cases where semen parameters are all within an acceptable range, but the male factor persists for reduced fertility [8]. When good quality semen doses are used, there is still around a 6% fertility variation at the farm level due to boar-related factors [9,10]. Therefore, it is crucial to identify these other boar-related factors that define subfertile boars, ideally before entering the breeding herd. This would allow pig producers an opportunity to enhance overall reproductive efficiency and improve the genetic dissemination of high merit animals.

Damaged sperm chromatin may impair the capability of the spermatozoa to fertilize an oocyte, decrease insemination success, cause abortion or fetal abnormalities and even reduce offspring vitality [11]. The sperm chromatin stability assay (SCSA) is a simple test that assesses sperm nuclear chromatin status by flow cytometry using acridine orange (AO) fluorochrome [12]. Boar sperm nuclear DNA is normally highly condensed and stable; the SCSA detects the abnormal chromatin structure and potential DNA damage that depends on the susceptibility of DNA to denaturation [13,14]. The results obtained from the SCSA are not correlated with other conventional semen parameters and can be considered an independent parameter [15].

Sperm quality can be influenced by multifactorial genetic factors [16] but also environmental factors such as stress and seasonality [17,18,19]. Especially in temperate climates, temperature and photoperiod show the most important effects on pig reproduction [20,21,22]. Previous studies devoted to evaluating the relationship between semen quality in boars and the influence of seasonality are ambiguous [23,24]. In industrial pig production (*Sus scrofa domestica*), a clear drop in male fertility has been observed in the warm summer months, possibly due to heat stress [25]. In industrial pig production, it is thought that sperm volume and concentration are lowest in spring; they gradually increase during the summer and reach a peak in late autumn [26]. Likewise, a daily variation in temperature greater than 10 °C has been identified as a depressor of sperm production [21]. In any case, animals reared in industrial AI studs are not completely exposed to the environmental conditions, but have heating, artificial light and fans so the season influence could be diminished.

Regarding molecular sperm quality, while boar-spermatozoa collected in spring–summer appeared to have a relatively higher percentage of spermatozoa with damaged nuclear DNA, a significant increase was only evident in fractionated ejaculates from two out of five boars [25]. Contrarily, Petrocelli et al. (2015) [27] reported that season, photoperiod or genetic line did not affect sperm nuclear DNA fragmentation. However, Peña et al. (2019) [14] described that a tropical summer induces nuclear DNA damage and reduces sperm concentration without depressing motility suggesting that traditional methods of evaluating sperm motility may not detect inherently compromised spermatozoa.

To the best of our knowledge, all the studies about photoperiod and sperm nuclear DNA fragmentation involved a limited number of boars and were performed with different analysis methods for DNA fragmentation index (DFI) determination or have been performed in the southern hemisphere with very different climates and seasonality to Spanish latitude. On the other hand, the present research is based on a retrospective study of a large number of ejaculates produced between 2014 and 2019 in different AI studs located in different parts of Spain, all of them analyzed with the same system. Besides, previous studies do not take into account other boar variables that could influence DFI, as is the case of the age of the boar [28].

The aim of this research was to study the effect of photoperiod, season and age of the boar on the sperm nuclear DNA fragmentation (measured by SCSA), a parameter that is not routinely evaluated in commercial AI boars.

## 2. Material and Methods

The study included approved methods and standard operating procedures for boar semen processing. The AI studs in this study complied with the Council Directive, 2008/120/EC outlining minimum standards for the protection of pigs and Directive, 2010/63/EU of the European Parliament and the Council of 22 September 2010 on the protection of animals used for scientific purposes.

### 2.1. Boars and AI Studs Involved

#### Barns and Animals

Total DNA fragmentation index (tDFI, %) of 1279 ejaculates from 372 different boars were studied. Data were collected in 6 different commercial AI studs located in 6 different provinces of Spain. AI studs were selected based on known common production procedures, similar housing systems and similar boar management and population. The size of the AI studs ranged from 80 to 300 boars. Boars were chosen randomly at the beginning of the study. They were always allocated in individual crates of >6 m^2^. All facilities had water available *ad libitum*, and animals were fed 1–2 times per day a total of 2.5–3.5 kg with a commercial boar feed (9.2 MJ/kg ME, 15.5% protein, 4.5% fiber, 12,500 IU/kg vit A and 170 IU/kg vit E). Although weather conditions differed among the stations, light exposure was controlled in all of them, with more than 150 lux at eye level during 14–16 h per day and limited contact with natural light. Temperatures inside the facilities were also controlled in all cases, ranging from 11 (coldest, in winter) to 28 °C (hottest, in summer). The birth date of each boar was recorded. The distribution (mean ± SD) of the age of the boars at collection was 19.6 ± 9.5 months.

We studied both the date of collection and the assumed date of testicular production of semen (defined as 50 days before collection; [28] in the testicles to classify in each one of the photoperiods. Dates of semen collection were chosen randomly from 10 March 2014 to 21 January 2019. The estimated dates of semen production in the testis were from 15 January 2014 to 26 November 2018. The studied 372 boars belonged to six pig breeds: Duroc (63), Landrace (39), Large White (21), Pietrain (156), synthetic hybrid lines (70) and York (23). They corresponded to several AI studs in Spain distributed as follows A (82), B (147), C (23), D (14), E (14) and F (92).

### 2.2. Photoperiod Classification 

The study involved boars with at least one valid DFI datum in both photoperiods (increasing photoperiod: from 21 December to 21 June; decreasing photoperiod: from 22 June to 20 December). As regards semen collection, there were 574 data from 372 boars for increasing photoperiod (1–3 data/boar) and 705 data from 372 boars for decreasing period (1–4 data/boar). As regards semen production, there were 688 data from 372 boars for increasing period (1–4 data/boar) and 591 data from 372 boars for decreasing photoperiod (1–3 data/boar) (Table 1 and Table 2).

In an additional approach, both photoperiods were divided in two subperiods: Minimum day length during increasing photoperiod phase (winter: from 21 December to 20 March), maximum day length during increasing photoperiod phase (spring: from 21 March to 20 June 20), maximum day length for decreasing photoperiod phase (summer: from 21 June to 20 September) and minimum day length for decreasing photoperiod phase (autumn: from 21 September to 20 December). Table 3 shows the number of data (*n*), number of individuals (*N*) and data per individual for each subperiod when considering the date of semen collection. Table 4 includes this information when considering the date of semen production.

### 2.3. Collection and Semen Sampling

In all cases, ejaculate collection was performed in a separate collection room. Double gloves and hygienic measures were taken with previous dry cleaning of the preputium and penis before collection. Ejaculate collection was performed with semiautomatic devices in all cases. All the ejaculate was collected, including rich and post-rich sperm fractions. Bacteriology was checked for each ejaculate in blood agar plates (37 °C during 48 h) with always less than 300 UFC/mL (data not shown).

Each AI stud collected semen samples for DFI evaluation regularly from the beginning of the trial until the finalization, every 12–16 weeks. All boars were sampled each time in each AI stud regardless of the sperm quality.

Ejaculates were taken to a production laboratory and samples were prediluted with TNE buffer (0.01 M Tris-HCl, 0.15 M NaCl and 1 mM disodium EDTA; pH 7.4) within 5 min after collection, aiming a concentration of 20 million sperm cells per mL. Diluted samples were stored immediately at −20 °C and then sent to the reference laboratory in styrofoam boxes with controlled freezing temperature in the following 24 h.

### 2.4. DNA Fragmentation

Sperm nuclear DNA damage was assessed by the SCSA as described by Evenson, (2016) [8]. Semen samples prediluted with TNE stored at −20 °C were sent to our laboratory at that temperature within 24 h. After arrival, samples were stored at −80 °C until further processing. For analysis, immediately after thawing an aliquot of 200 µL of each sample was treated with 400 µL of a solution containing 0.1% Triton X-100, 0.15 mol/L NaCl and 0.08 mol/L HCl pH 1.2. Then, 1.2 mL of staining buffer (6 µg/mL acridine orange, 100 mmol/L citric acid, 200 mmol/L Na_2_HPO_4_, 1 mmol/L disodium EDTA and 0.15 mol/L NaCl, pH 6.0) was mixed with the semen sample after 30 s. The resulting sample was analyzed by FACS (Becton Dickinson) using the software CellQuest v3 (Becton Dickinson), counting more than 2000 sperm cells (aiming 5000). Equivalent instrument settings were used for all samples following the procedure described by Martinez-Pastor et al. (2010) [12]. Histogram plots (total sperm cells vs. DFI) and DFI readings were calculated for each sperm cell. DFI is used to refer to the ratio between the red fluorescence and the total fluorescence (red + green) of individual spermatozoa. A series of threshold values are set for DFI to divide between moderate DFI (mDFI; cut-off at 0.25 DFI) and high DFI (hDFI; cut-off at 0.75 DFI) [12]. Spermatozoa that individually surpassed these values were classified as mDFI or hDFI as well. However, both moderate and high DNA fragmentation could lead to fertility problems while the cut-off is more theoretical than based on fertility consequences. Thus, total DFI (tDFI), the sum of mDFI plus hDFI was considered for this study.

### 2.5. Statistical Analyses 

All variables studied (tDFI, age at semen collection/production) were tested for normality distribution by the Shapiro–Wilks test. In case when significant departure from normality was detected a logarithmic transformation (e.g., LNtDFI) was used to correct this problem. A Pearson’s correlation (r) was run to assess the relationship between LNtDFI and age at semen collection/production. An ANCOVA (analysis of covariance) was run to determine the effect of photoperiod or subperiod (fixed effect) and boar (random effect) on LNtDFI after controlling for age at semen collection/production (covariate). This covariate is linearly related to the dependent variable (LNtDFI) and its inclusion into the analysis can increase the ability to detect differences between subperiods (independent variable). This ANCOVA was used to determine whether there are any statistically significant differences between the adjusted means of four subperiods. These adjusted means result from adjusting the crude photoperiod or subperiod means for chance imbalance in the distribution of the age among photoperiod or subperiods. In this way, the effect of age on LNtDFI was removed. *p*-values < 0.050 were considered as statistically significant. When a significant effect was detected, partial η was calculated as a measure of effect size (percentage of the variance in LNtDFI explained by the significant factor). Regression models were applied to the prediction of the evolution of tDFI as a function of the individual age. The coefficient of determination R^2^ measures the variability of LNtDFI explained by its relationship to age at semen collection/production; also, R^2^ is a measure of goodness of linear model fit.

## 3. Results

tDFI data ranged from 0.018% to 20.1%. As they significantly departed from normal distribution (Shapiro–Wilk test, *p* > 0.05), tDFI values were subjected to a logarithmic transformation that improved the results. The Q–Q (quantile–quantile) plot comparing the distribution of LNtDFI both for increasing and decreasing photoperiod against the normal distribution showed linearity of the points on the line y = x suggesting that LNtDFI was normally distributed (data not shown).

**Figure 1:**
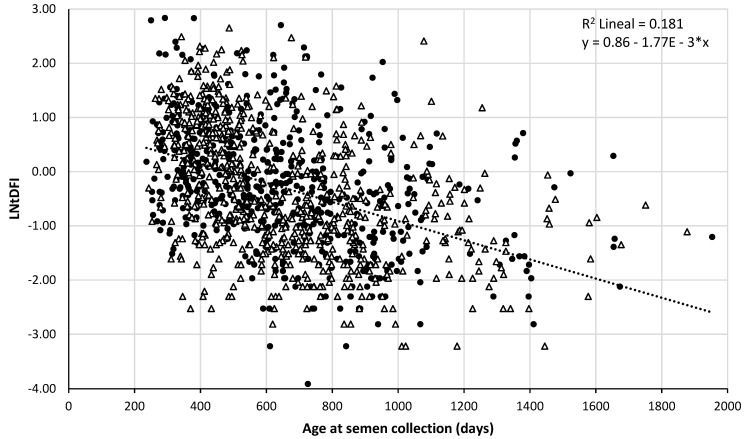
Relationship between sperm nuclear DNA fragmentation index and age of the boar at the collection of the ejaculate. Fragmentation is expressed as the LNtDFI and age of the boar is expressed in days. Data are represented according to its classification in increasing (⬤) or decreasing (△) photoperiod.

**Figure 2:**
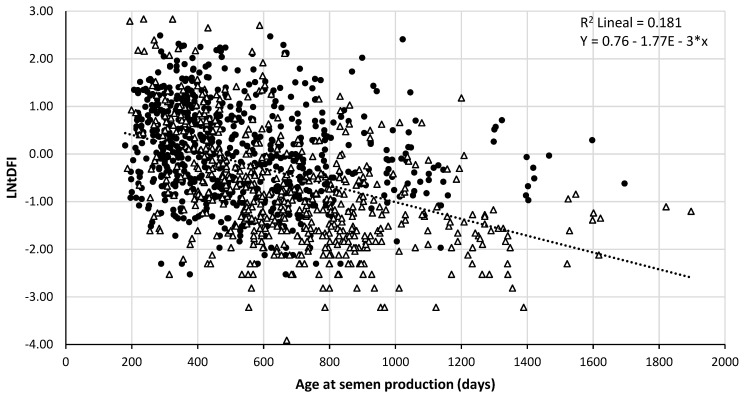
Relationship between sperm nuclear DNA fragmentation index and age of the boar at the expected time of production of the ejaculate in the testis. Fragmentation is expressed as the LNtDFI and age of the boar is expressed in days. Data are represented according to its classification in increasing (⬤) or decreasing (△) photoperiod.

The same, significant and negative r value was observed for both LNtDFI and age at semen collection and LNtDFI and age at semen production (r = −0.426, *p* < 0.001); when age increased, LNtDFI generally decreased. Linear regression of LNtDFI on age at semen collection/production explained 18.1% of observed LNtDFI variation (coefficient of determination R^2^ = 0.181). Figure 1 and Figure 2 plot the data of the 1279 ejaculates according to their LNtDFI and the age at collection or expected sperm production in the testis, respectively. As it is shown in the figures, some data change from one photoperiod to another depending on the date considered (collection or sperm production in the testis).

Two ANCOVA were run to determine the effect of photoperiod (fixed factor) on LNtDFI using the boar as a random effect and considering the age at semen collection or the expected age at semen production in the testicles as covariates. Table 1 shows the obtained results for LNtDFI (unadjusted and adjusted means) and age at semen collection in both photoperiods. LNtDFI values were evaluated for 654.25 days of boar age at semen collection (adjusted means). After controlling for age at semen collection, there was a statistically significant effect of boar on LNtDFI (F (371, 904) = 2.457; *p* < 0.0001, partial η^2^ = 0.502). However, there was no statistically significant difference in LNtDFI between photoperiods (F (1, 904) = 0.140; *p* = 0.708).

Table 2 shows the obtained results for LNtDFI (unadjusted and adjusted means) and age at expected semen production in the testis in both photoperiods. LNtDFI values were evaluated for 598.40 days of boar age at semen production (adjusted means). After controlling for age at semen production, similarly to age at collection, there was a statistically significant effect of male on LNtDFI (F (371, 904) = 2.221; *p* < 0.0001, partial η^2^ = 0.477). In addition, a statistically significant difference in LNtDFI between photoperiods was found (F (1, 904) = 34.349; *p* < 0.0001, partial η^2^ = 0.037). The decreasing photoperiod had the lowest LNtDFI (mean difference of 0.378; 95% CI: 0.251–0.505; *p* < 0.001).

After studying the effect of increasing or decreasing photoperiod, in an additional approach, it was evaluated whether the number of sunlight hours affects the seasonality of boar sperm DNA fragmentation. Data were classified in four seasons (winter, spring, summer and autumn). Two ANCOVA were run to determine the effect of seasons (fixed factor) on LNtDFI using the boar as a random effect and considering the age of the boar as a covariate. Table 3 shows the obtained results for LNtDFI (unadjusted and adjusted means) and age at semen collection in the seasons. LNtDFI values were evaluated in 654.25 days for semen production (adjusted means). After controlling for age at semen collection, there was a statistically significant effect on LNtDFI of both boar (F (371, 902) = 2.186; *p* < 0.0001, partial η^2^ = 0.473) and subperiod (F (3, 902) = 15.875; *p* < 0.0001, partial η^2^ = 0.050). The adjusted mean for LNtDFI was significantly lower in autumn than in the rest of subperiods. Additionally, the adjusted mean in summer and spring was significantly higher than in winter. No other significant differences among subperiods were found. 

Table 4 shows the obtained results for LNtDFI (unadjusted and adjusted means) and age at expected semen production in the testis in the different seasons. LNtDFI values were evaluated in 598.40 days for semen production (adjusted means). After controlling for age at semen production, similarly to age at collection, there was a statistically significant effect on LNtDFI of male (F (371, 902) = 2.217; *p* < 0.0001, partial η^2^ = 0.477) and subperiod (F (3, 902) = 49.609; *p* < 0.0001, partial η^2^ = 0.142). The adjusted mean for LNtDFI was significantly lower in autumn than in the rest of subperiods. Additionally, the adjusted mean in summer and winter was significantly higher than in spring. No other significant differences among subperiods were found. 

## 4. Discussion

The seasonality of reproduction is undisputable in intensive pig production in temperate climate countries [29]. Even when good quality semen doses are delivered from AI stations regarding sperm counts, sperm motility, sperm abnormalities and sperm longevity, results in farms can be impaired in certain periods of the year, related to temperature and photoperiod [30]. This fact has a strong farm effect [31] but also has a boar-related effect [10].

High DFI values have been related to reduced fertility, longer times to pregnancy and higher spontaneous miscarriage rates in humans [11,32] and it has shown a relation with pregnancy rate and total piglets born in boars [33]. Moreover, it is not related to other classic sperm quality parameters [8] and is a candidate to explain part of the boar-related effect of subfertility [9]. Therefore, the effect of photoperiod, season and age of the boar on DFI was evaluated.

Our first finding was that age of the boar was negatively correlated to tDFI.

Boars in AI studs are subjected to a high replacement rate due to the short pig generation interval and the competitive and fast genetic improvement of each new generation, surpassing the productive characteristics of its ancestors. This means that there is a rush in young animals to enter the AI studs and be trained to be included in the production of seminal doses at early ages. On the other hand, there are boars considered old (namely more than 3 years old), but still exploited due to the preference of local breeders or their outstanding genetic merit.

It is well known that ejaculate volumes and productivities are lower in young boars [28]. Semen volume increases from puberty until about 24 months age and remains relatively constant thereafter. Sperm concentration increases sharply from puberty to the first year of age, followed by a long-term moderate decrease until 3 years of age and it stabilizes thereafter [34]. Particularly for sperm fragmentation, [27] found that the age of the boar had a significant effect on DNA stability and chromatin structure. These authors found lower percentage of spermatozoa with DNA fragmentation in the group of boars aged 20–26 months old in comparison to the young animals (7–8 months old).

In the boar population of our study, tDFI ranged from 0.018 to 20.1% (LNtDFI: −4 to 3). The big number of randomly chosen ejaculates analyzed in the present study explains the bigger range of tDFI in comparison to other authors [27]. Didion et al. (2009) [7] proposed that boar spermatozoa with greater than 6% DFI might negatively affect both the farrowing rate and total piglets born. This threshold would correspond in our figure with LNtDFI values above 1.8 in which most of the boars are aged below 2 years. This result agrees with the timeline of the maturation of boar testicles described by Knecht et al., 2017 [31]. In addition, Boe-Hansen et al. (2008) [33] reported that the total number of piglets born for Hampshire, Landrace and Danish Large White boars was, respectively, 0.5, 0.7 and 0.9 piglets less per litter when the % tDFI values were above 2.1%. This cut-off would coincide approximately in Figure 1 with the LNtDFI of 1, above which most of the boars had in any case less than 2 years old.

The negative correlation of the tDFI and the age of collection and expected production of semen found in our study illustrates one potential hidden fertility troublesome related to the high turnover of boars at the AI studs. Accordingly, the average age of the boars studied at the moment of the ejaculate collection was 19.6 months. It is worth noting that the management of the inputs and deviate of boars in the AI stud could produce a bias in the interpretation of the results. The ejaculates of young boars that scored poorly in the conventional semen analysis were temporally discarded for seminal doses production and if the low productivity lasted for a certain time (namely 3–4 months) the animal was eventually culled. In parallel, the animals whose seminal doses produced proven low fertility results in repeated occasions were also removed from the AI stud. As DFI is correlated with low semen quality or poor fertility results [7,11,33], the AI stud management criteria were indirectly selecting boars that sustain sperm parameters above the standard and show good fertility results and would prevent boars with high tDFI values to get old in the AI studs.

The inclusion of individual boar as a random effect in the ANCOVA and the large number of boars and ejaculates studied ensure the soundness of the results obtained. Results show that the percentage of spermatozoa with fragmented nuclear DNA in young animals could decrease along with the aging of the boar being especially notorious until two years old. Therefore, these results support that the exploitation of young boars without testing their sperm nuclear DNA stability could bear to poor individual reproductive results, as previously suggested by [27]. Early diagnosis of sperm nuclear DNA fragmentation would allow for stricter selection and elimination of males with potential fertility disorders from breeding. Sperm DNA fragmentation could be a valuable prognostic tool to predict final fertility outcomes in pigs [9,35]. As it is shown in Figure 1, the equation obtained (y = 0.86 −1.77E − 3x) explains 18% of the variability of tDFI. According to this equation, tDFI would decrease due to aging of the boar 0.66% each year old.

Our second finding was that the photoperiod did not influence tDFI when the semen collection date was considered and age was included in the ANCOVA analysis as a covariate to control its effect (Table 1). Although, there was no statistically significant difference in LNtDFI between photoperiods of the collection date (*p* = 0.708), there was a statistically significant effect of boar on LNtDFI (*p* < 0.0001). These results show the large proportion of variability that is caused by the individual effect of boar. This high variability found among individuals agrees with other authors. For example, Didion et al. (2009) [7] found a range of %tDFI among 18 boars of 1.2–28.3%; Petrocelli et al. (2015) observed an individual variability in tDFI of 80% and Zasiadczyk et al. (2015) [25] found that boar was a significant source of variability in DNA fragmentation (*p* < 0.039). 

Although there were not statistically significant differences of photoperiod in tDFI considering the collection date, from a biological point of view it is reasonable to divide the photoperiod considering the date when the sperm of the ejaculates was produced in the testis. In boars, each spermatogenic cycle and the entire spermatogenic process lasts 8.6–9.0 and 40 days, respectively. The sperm transit through the epididymis takes approximately 10 days [36]. Therefore, in order to consider the period that influenced the samples collected, 50 days were subtracted from the date of collection to calculate the date of production (beginning of spermatogenesis).

Boar effect also influenced on LNtDFI when the results were analyzed considering the date of semen production in the testicles in photoperiod division (*p* < 0.0001). However, in this case, there were also statistically significant differences between the means of the photoperiods (*p* < 0.0001) (Table 2). On average tDFI was 1.46% higher in increasing photoperiod compared to decreasing photoperiod. Therefore, to put it briefly, the date of the collection had no significant effect on tDFI, while on the contrary, the expected semen production date in the testis did have a significant effect on tDFI.

These results agreed with the higher activity observed in late autumn and winter (decreasing photoperiod) in wild pigs, the ancestors of the *Sus scrofa domestica* [37]. Other authors found contradictory results of photoperiod in different characteristics of industrial boar production. For example, Knecht et al. (2013) [13] described that the average semen volume was lower during the increasing photoperiod and testicle activity was higher in late autumn; Rivera et al. (2005) [20] found that variations of the natural Mediterranean photoperiod do not induce substantial changes in overall semen-quality parameters (viability, morphological abnormalities and total motility); Sancho et al. (2004) [24] on the contrary, found that sperm quality of the boars decreased during decreasing photoperiods (concentration, volume and the number of semen doses) but no differences of motility and vitality nor in the osmotic resistance were detected; Sonderman and Luebbe (2008) [38] stated that the percentage of ejaculates discarded varied among seasons; and Rodriguez et al. (2017) [39] associated this seasonal variation with changes in photoperiod and heat stress during summer.

Finally, when the effect of seasons (winter, spring, summer and autumn) was assessed in an additional approach in the present research, a significant effect on boar sperm DFI was found not only considering the age at semen production in testicles, but also in semen collection. For both dates, the lowest tDFI values corresponded to minimum day length for decreasing the photoperiod phase (autumn), while the highest tDFI values were found in summer (maximum day length for decreasing photoperiod phase) (Table 3 and Table 4). In general, a sustained upward trend on tDFI was observed in winter, spring and summer, followed by a drop in Autumn. Furthermore, the season of semen production had a greater effect on fragmentation (partial η^2^ = 0.142) than the season of semen collection did (partial η^2^ = 0.050). This result points out to a direct effect of season on semen production that could consequently be detected 50 days later.

Previous studies show heterogeneous results of the effect of seasonality on DFI. However, it is difficult to compare the present research with the published literature because the techniques used to measure DFI are different and the number of boars previously under study is usually low. Zasiadczyk et al. (2015) [25] used the Neutral COMET Assay (Fraser et al., 2007) [40] to assess the seasonal variations in the DFI of semen from five mature polish large white boars. A lower DFI was observed during the autumn–winter period in comparison to spring–summer. However, the seasonal effect on DFI was less marked compared to the increase in sperm concentration and the number of spermatozoa found in the autumn–winter period. Petrocelli et al. 2015 [26] evaluated the effects of season, natural photoperiod and room temperature on semen collected from eight boars in Uruguay. DFI was measured with the sperm chromatin dispersion test (SCD) (Halomax, Halotech, Madrid, Spain). The SCD technique is a low-resolution technique, which counts a few sperms per sample, with low repeatability [41] and thus not comparable to SCSA [8]. Petrocelli et al. (2015) [26] did not find differences in DFI between seasons in agreement with other studies in different species [42]. However, in this study the ejaculates were preselected in the AI stud before the DFI analysis: A preliminary evaluation of the volume, subjective motility and number of spermatozoa was performed to score the ejaculates from 1 (worst) to 5 (best), and ejaculates scoring 3 or less were discarded. This could produce a bias in the study because the bad ejaculates according to these criteria were systematically discarded for the DFI test thus differences between seasons could be hidden. Peña et al. (2019) [14] evaluated the influence of Australian seasons (early dry, late dry and wet peak) on sperm DFI from five large white boars. BTS-diluted semen samples were purified by Percoll gradient centrifugation to discard possibly dead and damaged spermatozoa before being analyzed by TUNEL. Although TUNEL and SCSA do not detect the same DNA damage or weakness [13] their results can be considered equivalent [8]. However, the preselection of ejaculates by Percoll filtration could bias the results. In any case sperm DNA damage (%) was 16.1 ± 4.8 in peak wet, 1.0 ± 0.2 in early dry and 1.9 ± 0.5 in the late dry season. Thus, DNA fragmentation during the tropical summer (peak wet season) was 16-fold greater than during the early dry and nearly 9-fold greater than during the late dry season. DNA damage was attributed by the authors to the heat stress that also reduced concentration, but motility was not depressed. This research suggested that traditional methods of evaluating sperm motility might not detect inherently compromised spermatozoa.

Boars are particularly vulnerable to the effects of heat stress because their sweat glands are located deeper in the dermis and in subcutaneous tissue except for the eccrine type in the snout and dorsal nasal regions being inefficient at sweating [43,44]. In addition, the boar scrotum is not pendulous and boar spermatozoa tend to be more susceptible to temperature shock [45]. Heat stress produces higher rates of morphological abnormalities in spermatozoa [46] and a big temperature variation has proven to negatively affect the boars [21]. Although the temperature of the AI studs of the present research is controlled, it seems that the boars are not completely free from heat stress. On the other hand, all the AI studs studied in the present research have access to sunlight although it is limited, anyway, the animals could perceive the photoperiod. Thus, it seems that the higher percentage of sperm DNA fragmentation during increasing photoperiod and especially during summer could be related not only to the daily hours of sunlight but also to the residual heat stress of AI studs in Spain. 

## 5. Conclusions

Although there is a significant single boar effect in the tDFI occurrence, the boar age is negatively correlated with tDFI measured by SCSA. The development of mathematical models to predict the evolution of DFI of the individuals could reduce the premature discarding of boars and optimize the age of production in the AI studs. The systematic use of SCSA tests in the AI studs especially applied in semen samples from young animals that have been recently incorporated is an interesting strategy to improve the selection of males with disorders that may pose a potential subfertility.

Furthermore, while no differences were found in tDFI between photoperiods when the sperm collection date was evaluated, the percentage of spermatozoa with fragmented nuclear DNA was higher in semen produced in the testis during increasing photoperiod in comparison to decreasing photoperiod. On the other hand, for both dates, the lowest tDFI values corresponded to minimum day length for decreasing photoperiod phase (autumn), while the highest tDFI values were found in summer (maximum day length for decreasing photoperiod phase and maximum temperatures).

Temperature, humidity, hours of natural light and other factors could be involved in the seasonality of DFI. The effective control of the environmental conditions in the AI stud and the routine testing could palliate the negative effect of seasonality in male fertility. 

## Figures and Tables

**Table 1:** Means and variability of age at semen collection and unadjusted and adjusted means and variability for LNtDFI with age of semen production as covariate. LNtDFI values were evaluated in 654.25 days for semen production (adjusted means). *n*: number of data; SD: standard deviation; SE: standard error.

Photoperiod	*n*	Age of Semen Collection (Days)		LNtDFI (Unadjusted)		LNtDFI (Adjusted)	
		Mean	SD	Mean	SD	Mean	SE
Increasing	574	654.43	279.270	−0.2706	1.11866	−0.389	0.038
Decreasing	705	633.05	278.738	−0.3255	1.18808	−0.370	0.035

**Table 2:** Means and variability of age at expected semen production in testis, and unadjusted and adjusted means and variability for LNtDFI with age of semen production as a covariate. LNtDFI values were evaluated in 598.40 days for semen production (adjusted means). *n*: number of data; SD: standard deviation; SE: standard error.

Photoperiod	*n*	Age of Semen Production (Days)		LNtDFI (Unadjusted)		LNtDFI (Adjusted)	
		Mean	SD	Mean	SD	Mean	SE
Increasing	688	527.12	251.505	0.0931	0.989900	−0.206	0.039
Decreasing	591	681.30	286.310	−0.7602	1.169810	−0.584	0.043

**Table 3:** Means and variability of age at semen collection and the unadjusted and the adjusted means and variability for LNtDFI with age of semen production as covariate. LNtDFI values were evaluated in 654.25 days for semen production (adjusted means). *n*: number of data; *N*: number of individuals; SD: standard deviation; SE: standard error. Means with different superscript are different (*p* < 0.05).

Season	*n* (Data)	*N* (Individuals)	Data/Individual	Age of Semen Collection (Days)		LNtDFI (Unadjusted)		LNtDFI (Adjusted)	
				Mean	SD	Mean	SD	Mean	SE
Winter	352	324	(1–3)	735.70	14.668	−0.6366	1.12147	−0.403 ^a^	0.050
Spring	222	212	(1–2)	525.57	15.701	0.3082	0.83535	−0.352 ^b^	0.073
Summer	442	340	(1–4)	615.55	12.387	0.0159	1.09573	−0.187 ^b^	0.043
Autumn	263	262	(1–2)	718.75	18.283	−0.8991	1.11579	−0.690 ^c^	0.058

**Table 4:** Means and variability of age at expected semen production in testis, and unadjusted and adjusted means and variability for LNtDFI with age of semen production as covariate. LNtDFI values were evaluated in 598.40 days for semen production (adjusted means). *n*: number of data; *N*: number of individuals; SD: standard deviation; SE: standard error. Means with different superscript are different (*p* < 0.05).

Season	*n* (Data)	*N* (Individuals)	Data/Individual	Age of Semen Production (Days)		LNtDFI (Unadjusted)		LNtDFI (Adjusted)	
				Mean	Standard Deviation	Mean	Standard Deviation	Mean	Standard Error
Winter	315	280	(1–3)	523.71	14.506	0.1855	0.81631	−0.086 ^b^	0.056
Spring	362	297	(1–2)	528.54	12.980	0.0334	1.11823	−0.257 ^a^	0.046
Summer	151	148	(1–2)	550.97	21.229	0.2241	0.89280	0.072 ^b^	0.077
Autumn	451	340	(1–2)	722.42	13.233	−1.0864	1.05103	−0.813 ^c^	0.046

## Data Availability

Data supporting reported results can be sent to anyone interested by contacting the corresponding author.
